# Osteoblastic Cell Sheet Engineering Using P(VCL-HEMA)-Based Thermosensitive Hydrogels Doped with pVCL@Icariin Nanoparticles Obtained with Supercritical CO_2_-SAS

**DOI:** 10.3390/pharmaceutics16081063

**Published:** 2024-08-13

**Authors:** Rubén García-Sobrino, Isabel Casado-Losada, Carmen Caltagirone, Ana García-Crespo, Carolina García, Juan Rodríguez-Hernández, Helmut Reinecke, Alberto Gallardo, Carlos Elvira, Enrique Martínez-Campos

**Affiliations:** 1 Polymer Functionalization Group, Departamento de Química Macromolecular Aplicada, Instituto de Ciencia y Tecnología de Polímeros-Consejo Superior de Investigaciones Científicas (ICTP-CSIC) Calle Juan de la Cierva, n° 3, 28006 Madrid, Spain; ruben.sobrino@urjc.es (R.G.-S.); carolina@ictp.csic.es (C.G.); hreinecke@ictp.csic.es (H.R.);; 2 Group of Organic Synthesis and Bioevaluation, Instituto Pluridisciplinar, Universidad Complutense de Madrid (UCM), Associated Unit to the ICTP-IQM-CSIC, Paseo Juan XXIII, n° 1, 28040 Madrid, Spain; 3 Department of Applied Mathematics, Materials Science and Engineering and Electronic Technology, Universidad Rey Juan Carlos, Calle Tulipán s/n, 28933 Móstoles, Spain

**Keywords:** thermosensitive hydrogel, cell sheet engineering, bone differentiation, icariin, supercritical CO_2_ technology, drug delivery

## Abstract

New clinical strategies for treating severe bone and cartilage injuries are required, especially for use in combination with implant procedures. For this purpose, p(VCL-co-HEMA) thermosensitive hydrogels have been activated with icariin-loaded nanoparticles to be used as bone-cell-harvesting platforms. Supercritical CO_2_-SAS technology has been applied to encapsulate icariin, a small molecule that is involved in osteoblastic differentiation. Thus, physical-chemical analysis, including swelling and transmittance, showed the impact of HEMA groups in hydrogel composition. Moreover, icariin (ICA) release from p(VCL-co-HEMA) platforms, including pVCL@ICA nanoparticles, has been studied to evaluate their efficacy in relevant conditions. Finally, the thermosensitive hydrogels’ cell compatibility, transplant efficiency, and bone differentiation capacity were tested. This study identifies the optimal formulations for icariin-activated hydrogels for both control and HEMA formulations. Using this technique, osteoblastic sheets that were rich in collagen type I were successfully transplanted and recultivated, maintaining an optimal extracellular matrix (ECM) composition. These findings suggest a new cell-sheet-based therapy for bone regeneration purposes using customized and NP-activated pVCL-based cell platforms.

## 1. Introduction

Tissue engineering (TE) is based on the application of three fundamental pillars that form the TE triad: cells, biological factors, and biomaterials [1]. Controlling these features enables major tissue regeneration, even in circumstances when natural healing does not occur. A clear example related to bone regeneration is critical-sized defects arising as a consequence of an accident or resection process associated with other pathologies [2,3]. In these situations, common therapies aim to facilitate biological repair, and restoring function typically involves the use of an implantable device to promote bio-integration and tissue healing. Hence, controlling cell processes is crucial, not only to avoid immune responses and biomaterial rejection [4] but also to enhance cell adhesion, proliferation, differentiation, or other functions such as matrix secretion and maturation. This is essential to achieve clinical success, avoiding second surgeries or other potentially adverse outcomes. For this purpose, different scientific proposals have been developed in recent years with the aim of combating such deficiencies, for example, the use of injectable hydrogels for cartilage regeneration or the oral consumption of probiotics that stimulate the intestinal microbiota, which is closely related to bone metabolism [5,6]. Similarly, the development of printable bioinks or MOF systems that allow the production of systems with high precision in terms of design has also shown great potential for producing bioactive implantable systems [7,8].

Another powerful tool to improve these processes is proposed in this work, namely, the use of cell therapy to augment biological activity within the implant micro-environment. In particular, cell sheet engineering has proven its efficacy in accelerating injury regeneration through the application of 2D tissues with an extracellular matrix [9]. Moreover, their use can be combined with implants in both pre- and post-implantation scenarios. In this situation, the control of cell growth and differentiation in an in vitro setting becomes critical for graft survival and development, especially for autologous transplants. 

In the last few years, our group has developed a platform for cell sheet harvesting based on *N*-vinyl caprolactam (VCL)-derived hydrogels [10], which present thermosensitivity and a volume phase transition temperature (VPTT) that is close to the physiological temperature most suitable for their use in the biological range [11]. The thermosensitivity of this type of polymer is based on the balance of polymer–polymer versus polymer–water interactions. Below the transition temperature, VPTT, the latter are more important, and the network can be considered to be hydrophilic and to absorb a lot of water. Above the VPTT, on the other hand, polymer-polymer interactions are predominant; the network can be considered hydrophobic and contains little water, much less than below the transition temperature [12]. Aiming to serve as smart supports for cell culture manipulation, these VCL-based hydrogels also enable cell adhesion and proliferation, and, more importantly, they allow for the gentle detachment of cell monolayers with a controlled temperature decrease (below VPTT), facilitating direct cell sheet transplantation to a new surface in both flat and non-planar 2D cultures [10,11]. This phenomenon occurs based on the aforementioned change in the hydrophobic/hydrophilic character of the thermosensitive network, i.e., when the temperature is reduced below the physiological temperature, the hydrophilic character of the network allows for higher hydration of the chains, leading to an increase in the absorbed medium content and, thus, detaching the proliferating culture [11]. The described gentle detachment allows the researcher to maintain cell–cell and cell–ECM interactions, unlike other conventional and currently used methods, such as the use of mechanical agents or proteolytic enzymes (scrapers or trypsin, respectively) that damage the previously mentioned structure [13,14]. Also, these VCL hydrogels are characterized by extraordinary mechanical behavior, facilitating their easy handling. Similarly, they can be chemically functionalized by adding monofunctional-type methacrylates [15,16,17] to the polymerizable formulation, allowing the researcher to obtain intelligent polymeric supports with more accurate specific characteristics for different advanced therapies. 

Thermosensitive polymers could also be applied in the biomedical field as drug delivery carriers, due to their hydrated state and ability to change their swelling dynamics in response to temperature, i.e., the thermosensitivity of this family of materials provides an additional feature by which to adjust the dynamics of drug delivery [18]. In this sense, small molecules such as icariin (ICA) play an important role in the molecular cell differentiation pathways, since they can induce a synergistic effect with other master regulatory proteins, such as bone morphogenetic proteins (BMP). Icariin is directly involved in osteogenic differentiation, showing both in vitro and in vivo effects [19,20]. This research addresses the culture and manipulation of osteoblastic cells on VCL-based hydrogels. For this purpose, the hydrogel used in this study has been loaded with icariin (ICA) in order to release it in a sustained manner during culture. ICA is a previously described molecule with the purpose of stimulating the differentiation process in osteoblastic cultures. In this sense, it is known that sustained release can promote the therapeutic effect compared to uncontrolled release processes that release all the drug in short bursts and can even lead to adverse effects on the regeneration of the target tissue [21]. To prepare the icariin release system, supercritical CO_2_ clean technology is used to perform the encapsulation process of ICA in linear pVCL (pVCL@ICA). The design considers the incorporation of pVCL@ICA to the hydrogel, to achieve a double-release modulation. Supercritical CO_2_ technology allows the production of micro- and nano-sized particles with the possibility of pre-selecting both the drug and the polymer of interest. More specifically, the technique presented was that of supercritical CO_2_ using SAS (supercritical anti-solvent) technology. Briefly, the efficacy of this method is based on the excellent solubility of the supercritical CO_2_ fluid with the proposed solvent and the low affinity of the latter with the solute, causing its precipitation and the consequent encapsulation of the candidate drug [22], thereby promoting the sustained release of ICA.

Furthermore, given the polyol character of ICA and its low water solubility, the possible influence of the presence of -OH groups in the hydrogel on the modulation of release has been evaluated by incorporating hydroxyethyl methacrylate, HEMA, into the polymerization materials. The hypothesis is that in aqueous media there may be polar interactions between the OHs groups of the two structures. Likewise, pHEMA itself has previously shown interesting properties for bone tissue engineering developments, demonstrating optimal cytocompatibility with the relevant bone cell lines [23]. In the same way, functional chemical groups may have a strong influence on processes such as cell differentiation [18]. In the case of the hydroxyl groups, their presence has been shown to favor chondrogenic differentiation in human mesenchymal stem cells (hMSC) [24].

Therefore, and based on the aforementioned rationale, this work presents a combined approach to controlling biological behavior by functionalizing thermosensitive hydrogels with HEMA groups and the controlled release of pVCL@ICA nanoparticles (NPs) by SC-CO_2_. Thus, in the first part of the work, a complete study of the characteristics of both the network and the thermosensitive particles is detailed. In the second part, the impact of pVCL@ICA on the harvesting of osteoblastic cell sheets for use in applications related to bone tissue engineering is studied.

## 2. Materials and Methods

### 2.1. Materials

Icariin (ICA), azobis-isobutyronitrile (AIBN), N-vinyl caprolactam (VCL), 2-hydroxyethyl methacrylate (HEMA), ethylene glycol dimethacrylate (EGDMA), and 1-hydroxyl cyclohexyl phenyl ketone (HCPK) were purchased from Sigma-Aldrich, while 1,3-divinylimidazolidin-2-one (DVI) was supplied by BASF, and absolute ethanol (EtOH) and dimethyl sulfoxide (DMSO) were acquired from Scharlau. Finally, the CO_2_ bottles were supplied by Carburos Metálicos (Air Products Group).

For the biological studies, C2C12-GFP (CRL-1772; green fluorescence protein) and MC3T3-E1 (CRL-2593) were sourced from ATTC (Manassas, VI, USA). Furthermore, fetal bovine serum (FBS) was purchased from Thermo Scientific (Hyclone^®^, Thermo Scientific, Waltham, MA, USA). Dulbecco’s modified Eagle medium (DMEM) that is high in glucose (D6429), minimum essential medium (α-MEM), and antibiotics (penicillin, streptomycin, and G418) were sourced from Sigma. In addition, 12- and 24-well (both treated and non-treated) plates were purchased from Corning Costar (New York, NY, USA). Finally, phosphate buffer saline, or PBS, was obtained from Gibco.

### 2.2. Materials Preparation

#### 2.2.1. Synthesis of Thermosensitive Hydrogels

The VCL hydrogels used in this work were synthesized by radical one-step photopolymerization, using HCPK as a photoinitiator at 0.5 wt.% with respect to the rest of the formulation. In turn, two crosslinkers were used, EGDMA and DVI, in molar percentages of 1.38 and 0.13 mol%, respectively, with respect to the molar content of VCL. To study the influence of HEMA, different molar percentages with respect to the VCL content were studied (0, to act as a control, 1.0, 2.9, 5.8, 8.6, and 11.6%).

Prior to the light curing process, the mixtures were bubbled with N_2_ to remove any possible trapped oxygen. Then, the formulations were transferred to polyethylene-coated polystyrene molds, separated by 0.3 mm of commercial silicone, using commercial syringes and needles. Regarding the photo-curing process, for 40 min, a wavelength of 365 nm was applied to the samples from a UV chamber (model CL-1000L, 230 V) with five lamps inside, generating data close to 3500 μW/cm^2^.

After the photopolymerization process, the hydrogels were removed from the molds and immersed numerous times in distilled water (diH_2_O) to remove any residual and unpolymerized formulation. Likewise, the samples were stored in EtOH (70%) at 4 °C and washed 24 h prior to any experiment in diH_2_O or PBS to ensure that the EtOH was completely removed.

For the VCL-hydrogels loaded with NPs as described below, 0.28 wt.% of each selected nanoparticle was included in the formulation.

#### 2.2.2. Synthesis of Linear pVCL

pVCL linear polymer was obtained by VCL homopolymerization in ethanol ([VCL] = 1 mol/L), with AIBN as the radical thermal initiator ([AIBN] = 1.5 × 10^−2^ mol/L). The mixture was left at 60 °C in an oven for 24 h for thermal polymerization. The polymer was then subjected to a purification process by dialysis with diH_2_O, using dialysis membranes of 3.5 kDa molecular weight, to be subsequently lyophilized.

#### 2.2.3. Fabrication of p-VCL@ICA Nanoparticles Using CO_2_ Supercritical SAS Technology

Linear pVCL was used as the matrix in this section. The particles were formed with the SAS R100 pilot plant from Thar Technologies^®^ (Thar Technologies, Inc., Pittsburgh, PA, USA). Different procedures were used to obtain the particles, changing the following variables: temperature, pressure, and solute-solvent concentration. A polymer-drug solution flow of 1 mL/min was kept constant in all the experiments. Once the desired temperature was reached, CO_2_ was pumped at the desired flow rate through the high-pressure pump until the pressure and temperature stabilized in the SAS reactor. The sample solution (pVCL and ICA) was then pumped into the reactor through its nozzle. A total of 20 mL of the solution was exposed to the CO_2_ pumping process to obtain particles. Once all that volume had passed, the flow of CO_2_ was maintained for a certain time, a period known as washing time, to eliminate the residual solvent. Finally, depressurization was performed slowly and gradually, and the particles were recovered from both the walls and the bottom of the container basket. Hence, the solvent dissolved in the supercritical fluid due to its miscibility, causing nanoparticle (NP) formation by precipitation, after which the particles were collected in the SAS reactor basket. 

### 2.3. Methods for Material Characterization

#### 2.3.1. Hydrogel Characterization

The chemical characterization of networks was analyzed by spectroscopy (FTIR-ATR). FTIR measurements were carried out on a Perkin Elmer Spectrum One spectrometer using an FTIR device in the range of 400–4000 cm^−1^ and using the attenuated total reflectance (ATR) mode, with a resolution of 2 cm^−1^.

To determine the degree of swelling of the hydrogels vs. temperature, samples of 12 mm in diameter were obtained at 20 °C by die-cutting the hydrogels in the swollen state. The swelling ratio (S) was defined as the weight of the aqueous medium per dry network (Equation (1)), where W_h_ is the swollen weight and W_d_ is the dried weight. Samples were left for 24 h between measurements to ensure an equilibrium state.

S = (W_h_ − W_d_)/W_d_ (1)

This study was performed in PBS in triplicate. Finally, the VPTT was calculated from the point of intersection of the plateau line of the onset with the slope of the curve of the graph [19].

Turbidimetry measurements were carried out by measuring the absorbance at 600 nm as a function of the temperature, using a UV–Vis spectrophotometer coupled with a temperature controller (Cary WinUV, Agilent Technologies, Inc., Santa Clara, CA, USA). PBS was used as a medium and the rate of heating was 1 °C/min. The transition temperature, which has been labeled in this work as the optical transition temperature (T_OT_) to differentiate it from the cloud point temperature (T_CP_) characteristic of linear chains, was calculated from the onset of the plot [25]. Polypropylene (PP) sheets were used as supports to maintain the sample’s position stably upon heating.

#### 2.3.2. pVCL@ICA NPs Characterization

To evaluate the encapsulation efficiency percentage (% EE) and loading of ICA after the SAS process (see Equation (2)), UV-Vis spectroscopy was used at 270 nm (Specord 205) with an ICA solution in EtOH at a concentration of 10^−4^ mol/L. A wavelength of 270 nm was chosen because it is the most characteristic absorption peak for ICA in the range being evaluated.

(2) $$EE(\%)=\frac{\left(\frac{m_{ICA}}{m_{pVCL}}\right)experimental}{\left(\frac{m_{ICA}}{m_{pVCL}}\right)theoretical}\;\times \;100$$

Nuclear magnetic resonance (^1^H-NMR) spectra were recorded at 25 °C in 10% (*w*/*v*) CDCl_3_ solutions with a Varian Gemini XLR at 300 MHz under standard conditions. 

The molecular weight average, molecular number average (Mw and Mn, respectively), and dispersity index of the samples were measured by size exclusion chromatography (SEC) with Waters 1515 Isocratic HPLC pump equipment (Waters Cromatografía, S.A., Cerdanyola del Vallès, Spain), calibrated with narrow molecular weight distribution to polystyrene standards. The data were recorded at 70 °C in 5% (*w*/*v*) DMF and 1 mL/min flow (Ultrastyragel columns of 500, 104, and 105 Å). 

The thermal transition (Tg) of the samples was analyzed by a DSC (Perkin-Elmer DSC-7) connected to a Perkin Elmer Intracooler 2P. The polymer was heated from 35 °C to 235 °C at 20 °C/min, with a N_2_ atmosphere as the purging gas. 

To characterize the drug carrier’s diameter and dispersion, a Phillips scanning electron microscope (ESEM XL30, North Billerica, MA, USA) was used with an accelerating voltage of 25 kV and employing ImageJ software (1.54f, National Institute of Health, Bethesda, MD, USA) to measure the size and distance values.

### 2.4. In Vitro Drug Release

A study of the in vitro release of ICA at 37 °C and PBS from NPs and NP-loaded hydrogels was carried out. In each case, the experiments were performed in triplicate, and a 10 mL volume of PBS was used; at appropriate times, a 1 mL aliquot was removed for the purposes of measurement and replaced with fresh PBS. The mass of ICA for each measurement was obtained by UV-Vis spectroscopy at 320 nm (with a plate reader (Synergy HT, Brotek)), using this calibration line: mICA(mg) = A(320)/1.137. In the case of NPs, 2 mg of NPs (5, 10, or 20) was dispersed in 10 mL of PBS. In the case of hydrogels, a piece 10 mm in diameter was die-cut in the swollen state at 37 °C and immersed in PBS.

### 2.5. Biological Evaluation

#### 2.5.1. Preparation of Materials before Cell Evaluation

Round-shaped hydrogels with diameters of 20 mm were cut in PBS at 37 °C. Before cell culture, the hydrogels were extensively washed and sterilized. Briefly, the hydrogels were first washed in an EtOH 70% solution for 10 min for three consecutive rounds. After that, a PBS solution was used, repeating the same procedure (×3, 10 min). Then, the hydrogels in PBS were placed under a germicide UV light for 20 min on each side, with a subsequent wash in DMEM. Eventually, the hydrogels were incubated overnight at 37 °C and 5% CO_2_ in complete DMEM, supplemented with 10% FBS and 1% antibiotics (penicillin/streptomycin, 100 U/mL). 

For the evaluation of pVCL@ICA NP cytocompatibility, the samples were sterilized by UV radiation for 2 h. Finally, for the tests with loaded NP-ICA hydrogels, sterilization was reduced to avoid the releasing of the molecule by successive washes. Therefore, only two ethanol 70% washes and PBS washes of 10 min each at 60 °C and UV (40 min) irradiation were performed. Additionally, complete DMEM was added to the impregnated samples overnight, along with proteins that facilitate cell adhesion.

#### 2.5.2. Cell Culture

Previously, the autofluorescent C2C12–GFP mouse premyoblastic cell line was genetically modified to achieve the constitutive expression of GFP (green fluorescent protein) with a lentiviral treatment. In this work, the cell line was cultured in complete DMEM in a humidified 5% CO_2_ atmosphere at 37 °C, and cell passaging was performed at a 90% confluence. For hydrogel evaluation, the cell density when seeded was 4 × 10^4^ cells/cm^2^. For cell imaging, an inverted fluorescence microscope (Olympus IX51, Olympus Evident, Barcelona, Spain) with a FITC filter (λ_ex_/λ_em_ 590/530 nm) was used.

In contrast, for the analysis of bone tissue differentiation, the MC3T3–E1 mouse preosteoblastic cell line was used. First, for the cell proliferation and transplant efficacy analysis, cells were seeded at 4 × 10^4^ cells/cm^2^ using a proliferation medium (α–MEM with 10% FBS and 1% of PS). To induce bone differentiation over the samples, two steps were required: first, the cells were seeded in a proliferation medium at a density of 4 × 10^4^ cells/cm^2^ and incubated for 4 days. Subsequently, the hydrogels were cultured for 28 days in a differentiation medium (α-MEM with 10% FBS and 1% of PS) supplemented with 10% β-glycerol phosphate (100 mM), 1% ascorbic acid, and 0.1% of dexamethasone, changing the media every 72 h. Finally, 16-millimeter-diameter pVCL hydrogels activated with pVCL@ICA NPs were seeded with the MC3T3–E1 cell line, with a density of 3 × 10^4^ cells/cm^2^, in a proliferation medium for 6 days; after that, the hydrogels were maintained in the differentiation medium as described earlier for 8 additional days. After transplantation (day 14), the cell cultures were maintained in the differentiation medium for 8 days before evaluation. All these studies were carried out at 37 °C and 5% CO_2_, performing cell passaging at 90% confluence.

#### 2.5.3. Methodology for Thermal Cell Sheet Detachment

As an initial cytocompatibility and transplant efficacy study, the C2C12–GFP cell line was used. For this goal, premyoblastic cultures growing on hydrogels for 72 h were transplanted using a controlled temperature decrease to a new well of a treated TCP. To maintain a humidified surface during this process, 10% of the total culture volume of fresh culture media was added to a new TCP well. Then, the cultured hydrogels were placed upside down into this new TCP well, and, immediately, 40% of the total culture volume was added. For 40 min, the hydrogels were incubated at 15–20 °C, adding 50% of culture media volume after 20 min. The hydrogels were finally removed, then the transplants in a new TCP well were reincubated under standard conditions, as described earlier.

#### 2.5.4. Metabolic Activity and Alamar Blue Assay

An Alamar Blue assay (Biosource, CA, USA) was used to analyze mitochondrial metabolic activity, following the manufacturer’s instructions. Briefly, this scalable and non-toxic procedure estimates cell viability using the reducing power of cell culture. For this goal, 10% of the culture volume of Alamar Blue dye was added to the cell culture and then incubated for 90 min. Fluorescence readings taken in triplicate (λ_ex_/λ_em_ 535/590 nm) were obtained using a multiwell plate reader (Synergy HT, BioTek, Winooski, VT, USA). 

#### 2.5.5. Collagen Secretion and PicroSirius Red Staining

In this work, collagen type I was selected as a bone differentiation marker during osteoblastogenesis. PicroSirius Red staining (Abcam, Cambridge Biomedical Campus, Cambridge, UK) was used to analyze collagen secretion to the extracellular matrix in osteoblastic cultures. Sample types were taken in triplicate and fixed for 15 min with 4% paraformaldehyde, then washed with PBS, and finally incubated for 30 min with the PicroSirius Red Stain Kit. Before imaging, washing with 0.5% acetic acid was performed to remove the excess dye. To allow quantification, a solution of NaOH 0.3 M/MeOH (1:1) was applied to each sample. Finally, the absorbance was measured at 540 nm using a microplate reader (Synergy HT, BioTek, Winooski, VT, USA).

#### 2.5.6. Statistical Analysis

The results were compared to demonstrate the differences between studies. Statistical analyses were performed using Student’s *t*-tests with GraphPad 4 software. Significant differences were indicated as follows: * (*p* ≤ 0.05), ** (*p* ≤ 0.01), and *** (*p* ≤ 0.001). 

## 3. Results and Discussion

**Figure 1:**
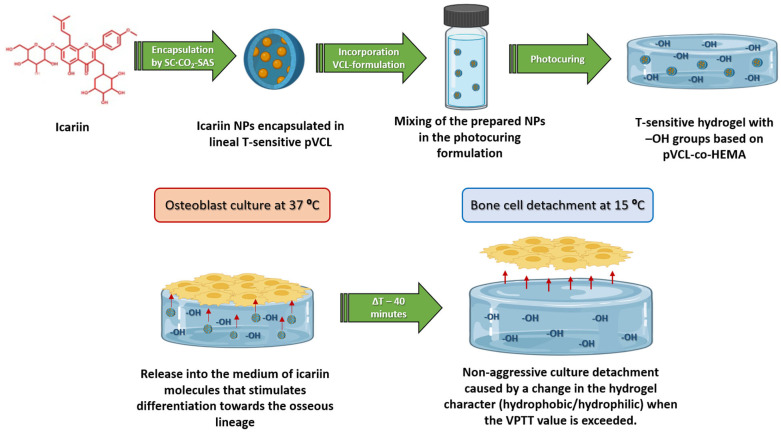
Scheme of the proposal evaluated in this work.

Figure 1 shows a representative scheme of the strategy evaluated in this work. As mentioned in the Introduction, VCL-based thermosensitive hydrogels have previously been reported to exhibit efficient cell adhesion and proliferation, as well as cell detachment upon decreasing the temperature in flat and non-planar 2D cultures [10,11]. In this work, ICA has been incorporated to modulate the differentiation and to evaluate the overall performance of the hydrogel in terms of adhesion, proliferation, differentiation, and detachment with osteoblast cultures. The dosage of specific biological factors, such as ICA, to stimulate bone tissue differentiation processes is crucial for therapeutic applications. 

### 3.1. Preparation, Characterization, and Cell Response Study of OH-Functionalized Hydrogels

As indicated in Figure 1, the ultimate goal of the study is to load ICA into both hydrogels without OH groups and hydrogels functionalized with different molar percentages of OH groups. Hydrogels functionalized with OH groups were prepared by including HEMA in the photocurable formulation. Before loading these hydrogel platforms with ICA, a study of these functionalized systems was performed. In addition to a control hydrogel without HEMA (labeled 0−HEMA), five hydrogels with varying amounts of HEMA from 1.0 to 11.8 mol% (labeled X−HEMA, where X is the nominal molar percentage of HEMA) were prepared by photocuring (see the experimental section, above), as shown in Table 1. 

Hydrogel formation by photocuring was evaluated and demonstrated using FTIR−ATR, as shown in previous works [11,16]. The incorporation of HEMA into the polymeric chains of the network was also qualitatively demonstrated by FTIR-ATR. A figure showing the spectra of the 0, 5.8, and 11.8−HEMA samples, after residual extraction in water and after drying, has been included in Table 1. At approximately 1720 cm^−1^, an increasingly intense signal was recorded as the percentage of copolymer that was associated with the vibrational tension of the carboxyl group (C=O) present in the ester of the HEMA comonomer increased, thus demonstrating in a qualitative form the presence of HEMA in the hydrogels. In addition, no peaks associated with the C=C group of the monomers, centered at 1655 cm^−1^, were observed [11].

It has previously been reported that control hydrogels without HEMA exhibit thermosensitivity and a transition temperature (VPTT) in the range of 33–35 °C [11], as well as a large increase in swelling with decreasing temperature (a more than 10-fold increase in the water content from 37 to 10 °C). As the objective of these platforms using HEMA is not only to modulate the interaction with ICA but also to maintain the performance in culture and in non-aggressive detachment, it is essential to study the influence of HEMA on thermosensitivity (both VPTT and swelling differences) and the resulting cell response. 

The smart capability mentioned with regard to thermosensitive networks implies the capacity of the hydrogel to drastically and reversibly modulate its structure as a function of temperature, causing variations in terms of swelling that also imply modifications at mechanical or transmittance levels [26]. At temperatures above the transition value, the chains involved exhibit a hydrophobic behavior in which the interaction between the hydrophobic segments of the system prevails. However, at temperatures below the transition value, the polymeric chain experiences an increase in its hydrophilic character; i.e., in this temperature range, the segments of the chain present a preferential interaction with the surrounding hydrated medium, causing a higher swelling capacity [12]. In terms of transmittance, this situation can cause the rearrangement of the chains during the process of partial expulsion of the medium, significantly reducing the transmittance of the system [27]. Hydrogels with sensitivity to different stimuli, such as micro- and nanoscale conducting hydrogels that are based on polymers conjugated and/or doped with metal particles or carbon derivatives, such as nanotubes and graphene, are now available [28,29]. In this sense, their properties related to self-healing or color sensitivity are far from the objective of non-aggressive cell culture detachment provided by the thermosensitive systems presented in this work.

Already, in the pre-study washes, it can be seen at a glance that the more HEMA the formulation contains, the less the swelling at low temperatures. Furthermore, HEMA−functionalized hydrogels gradually become stiffer by increasing the HEMA content. On the one hand, the thermosensitivity was studied gravimetrically, using PBS as the medium (pH 7.4). The figure in Table 1 shows the variation of the swelling ratio (S) in PBS as a function of temperature, which demonstrates the thermosensitivity of all samples, i.e., the systems below the VPTT value exhibit a higher absorption capacity of aqueous medium with respect to temperatures above the transition value. Regarding HEMA’s influence on swelling capability, the more HEMA is contained in the formulation, the smaller the swelling increase below the VPTT (see the values at 10 and 37 °C in Table 1). That is, HEMA contributes hydrophobically to the system. This finding is in agreement with the low solubility of pHEMA in water [30]. It should be noted that all the proposed hydrogels showed a hydrophobic character at a physiological temperature (37 °C), allowing a priori cell adhesion and proliferation. On the other hand, in terms of transmittance, thermosensitivity was also observed for all the proposed systems, where the difference in transmittance varies with temperature, with this variation being smaller as the molar content of HEMA increases, perhaps due to the decrease in thermosensitivity described above (the study showing all the temperature ranges is given in Supplementary Figure S1). Likewise, when analyzing the transmittance of all samples at 37 °C, as summarized in Table 1, it can be observed that the presence of HEMA units in the formulation favors a higher transmittance of the systems (exceeding 70% transmittance in samples with HEMA values of higher than 5.8 mol%), an increase that would facilitate the analysis of in vitro cultures, allowing a reduction in their manipulation during cell monitoring processes.

Finally, concerning the described transition value, VPTT, HEMA has hardly any influence on this parameter, despite previous works regarding linear poly (NIPAm-co-HEMA) copolymers showing a clear decrease in LCST with the incorporation of HEMA [31]. This event, already described previously in detail [17], could be explained based on the high difference in reactivity of the precursors involved (methacrylate vs. vinyl lactam). The first one showing superiority, in terms of kinetic reactivity [32], generates very rich regions of HEMA until its consumption. Hence, the growth process would be based on pVCL, which implies a heterogeneous process of polymerization. This heterogeneity in terms of distribution may explain the slight influence of the copolymer in the cited transition values.

In addition to studying the influence of HEMA on thermosensitivity, it is necessary to analyze its influence on cell culture. In previous works, the control network 0-HEMA has shown cell adhesion and proliferation capacity at 37 °C. Furthermore, as was mentioned previously, with a controlled temperature decrease (15–20 °C), the proliferated cell monolayer on the hydrogel surface detaches efficiently, keeping the cell–cell and cell–ECM interactions intact. 

**Figure 2:**
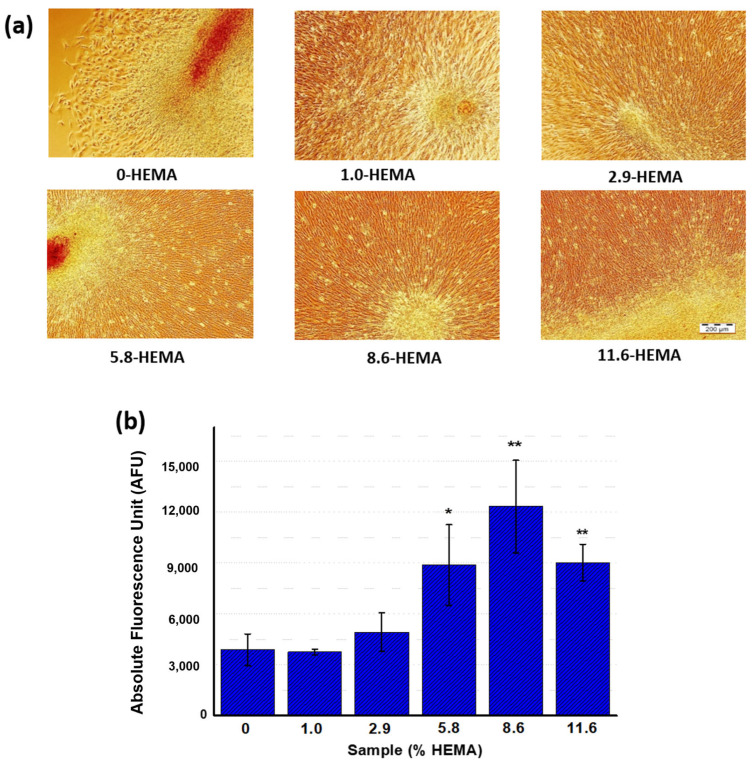
(**a**) Bright-field images of C2C12-GFP premyoblastic cell transplants 24 h after the cell transplant stage; (scale bar: 200 µm). (**b**) Metabolic activity (Alamar Blue) of cell transplants at 24 h. Significant differences were indicated as follows: * (*p* ≤ 0.05) and ** (*p* ≤ 0.01).

To analyze the cytocompatibility effect of HEMA in VCL-hydrogels, the cell proliferation capacity of the X−HEMA hydrogels was assessed. Hence, the premyoblastic and autofluorescent C2C12−GFP cell line was seeded on the hydrogels. As shown in Supplementary Figure S2 (72 h after the seeding stage), all surfaces showed attached cells proliferating over their surfaces, with elongated morphologies and no signs of cytotoxicity, demonstrating, therefore, their cytocompatibility at early stages. In the same way, it was a priority to evaluate how the presence of HEMA itself, and the fact that there is a comparable decrease in the increase in swelling as the temperature decreases, exhibited in a composition-dependent manner, influences the cell culture and detachment performance. At this point, after 72 h of the seeding process had passed, the cell monolayers, which were already at confluence, were transplanted upon temperature decrease (15–20 °C for 40 min) according to the process optimized in previous works [10,11] and described in Section 2.5.3 of this manuscript. Hereafter, all samples showed transplant capacity, and premyoblastic cell cultures that had detached from the X−HEMA hydrogels were observed 24 h after the transplant process (Figure 2a). To quantify transplant culture viability, the metabolic activity was measured using the Alamar Blue reaction. The results showed similarities in terms of the metabolic activity of the transplanted culture, up to 2.9−HEMA (Figure 2b). Significant differences in comparison to the control hydrogel were observed from 5.8-HEMA upward, indicating that these transplants presented an increased cell activity. This increased efficacy of the X−HEMA hydrogels can be attributed to the effect of HEMA groups in terms of early cell adhesion and proliferation, instead of an increase in transplant capacity, since the cell detachment efficiency upon lowering the temperature has been associated with the abrupt change in swelling, and this change decreases with increasing HEMA [13]. It must be noted that the maximum metabolic activity registered was found to be at a percentage of 8.6−HEMA. 

In a parallel approach, bone tissue differentiation and extracellular matrix (ECM) analysis were carried out using a preosteoblastic MC3T3−E1 model over PVCL−HEMA hydrogels (see Supplementary Figure S3). Unlike the results published by O. Lishchynskyi et al. [33], whose smart support is based on a smart coating on poly (di(ethylene glycol)methyl ether methacrylate (POEGMA) glass, an element with a chemical structure similar to pHEMA, the MC3T3−E1 line did not exhibit signs of cytotoxicity on our hydrogels, allowing cell proliferation as described in other works using pHEMA with bone lines [23]. Regarding the long-term experiment (30 days), all the hydrogels supported osteoblastic proliferation and differentiation, showing a collagen I secretion end time (see Supplementary Figure S4a). In contrast, no differences based on their HEMA content were detected (Supplementary Figure S4b), demonstrating adequate osteoblastic behavior over all pVCL hydrogels. These results support the application of these hydrogels as drug-loading platforms for advanced therapies in bone regenerative medicine.

In summary, it seems that the incorporation of -OH groups in the aforementioned VCL networks has shown a clear influence on the different properties of the hydrogel that needs to be addressed. On the one hand, at the physicochemical level, the networks, besides showing lower swelling capacity with the presence of HEMA in the formulation, have shown higher transparency, which is of interest in cell monitoring processes. On the other hand, regarding the in vitro studies, although all the proposed systems showed a cell differentiation capacity regarding bone lineage, the presence of HEMA in the formulation did not significantly improve this behaviour; however, systems that were functionalized with HEMA, especially at 8.6 mol%, showed a better capacity for non-aggressive cell detachment after a controlled decrease in temperature (also evaluated with the MC3T3-E1 cell line, see Supplementary Figure S5a,b, where the study of proliferation from dsDNA quantification is also included). According to the aforementioned findings, and in a comparative study, 0−HEMA (a control without HEMA) and 8.6−HEMA hydrogels were chosen to fabricate the complete constructs and study the influence of HEMA on the process. With 8.6−HEMA, this unit already has a clear influence on the metabolic activity in the study of cell detachment capacity.

### 3.2. Encapsulation of Icariin in Linear pVCL by Supercritical CO_2_-SAS Technology 

This work is based on the encapsulation of icariin molecules (ICA) by supercritical CO2-SAS technology in a system that is compatible with the previously developed VCL-hydrogels. For these purposes, pVCL nanoparticles with encapsulated ICA were prepared using linear pVCL, produced as indicated in the experimental section (Section 2.2.2). The linear thermosensitive polymer was characterized by ^1^H−NMR in CDCl_3_ (see Supplementary Figure S6) and GPC analysis, whereby an average molecular weight of 240 kDa and a dispersity index of 2.1 were obtained. 

Subsequently, concerning process optimization to fabricate pVCL@ICA NPs, the conditions proposed by V. Prosapio et al. [34] were used as guidelines. The constant variables were polymer–ICA, with a 1 mg/mL DMSO flow rate and 30 mg/mL CO_2_ flow rate. In contrast, three parameters of fabrication were optimized: temperature, pressure and solute–solvent ratio, as summarized in Table 2.

**Figure 3:**
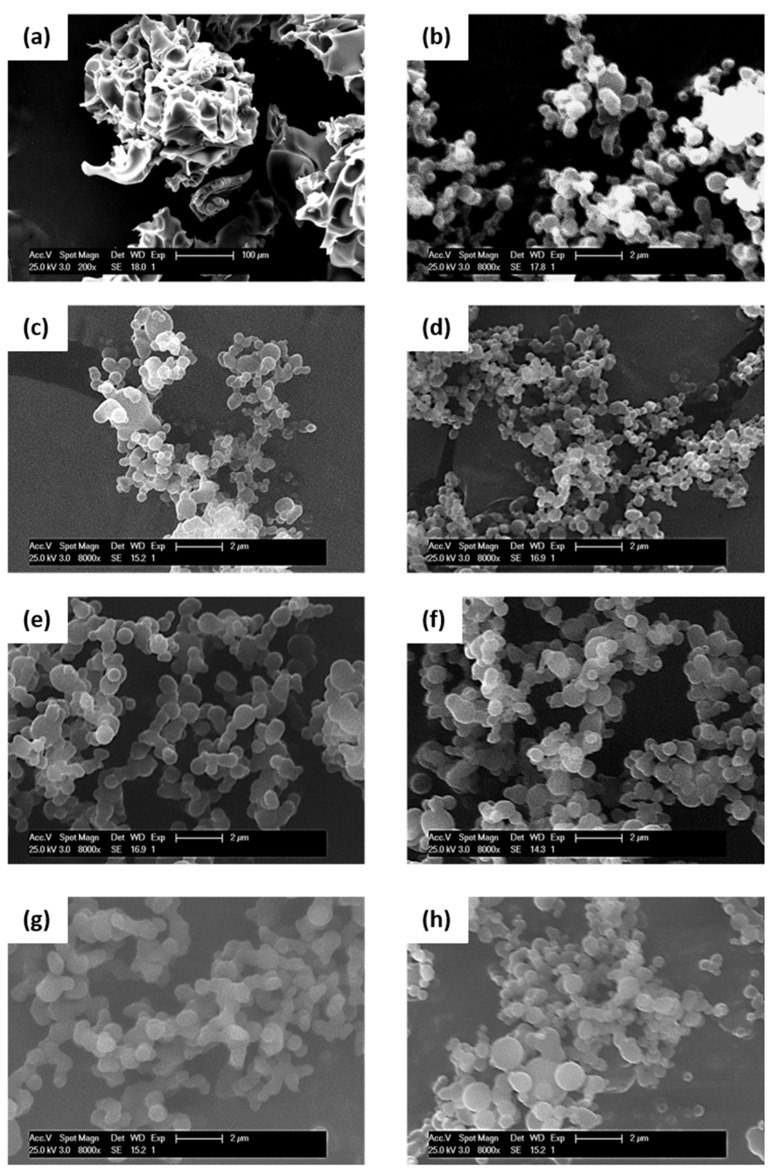
SEM micrograph evaluation: temperature optimization process at 40 (**a**) and 35 °C (**b**). Pressure optimization process at 100 (**c**) and 200 bar (**d**). pVCL-ICA concentrations at 20 (**e**) and 30 mg/mL (**f**). ICA load at 5 (**g**) and 20 wt.% (**h**). The scale bar for (**a**) is 10 µm and (**b**–**h**) is 2 µm.

Regarding temperature optimization, a temperature of 40 °C was first evaluated for encapsulation, according to the work of Abuzar et al. [35]. NPs that were synthesized at 40 °C did not acquire the desired spherical morphology but, rather, formed porous aggregates (Figure 3a). However, at 35 °C, it can be observed that a spherical shape was obtained with diameters of around 540 (see Figure 3b). Therefore, 35 °C was the selected temperature condition to continue. This finding is in line with the work of Zahran, R. et al. [36], wherein the influence of the process temperature on the increased DMSO miscibility in supercritical CO_2_ was discussed.

Secondly, the pressure value was optimized, maintaining the temperature at a constant 35 °C. The applied pressure varied between 100, 150, and 200 bar, obtaining, at 100 and 150 bar, particles with a spherical morphology and similar diameter size at 500–520 nm (see Figure 3c). At 200 bar, the NPs were smaller, with a diameter of approximately 390 nm, and were formed with higher heterogeneous distribution values that were associated with possible aggregates (see Figure 3d). Based on the above results, the feasible pressure range for the process seems to be between 100 and 150 bar. In order to reduce the variables, the selected pressure was 100 bars. As mentioned previously, Table 2 also shows the Tg values that were obtained, along with the encapsulation efficiency (EE), evaluated using a solution of ICA in EtOH at a concentration of 10^−4^ M.

After evaluating and selecting the optimum values of temperature and pressure for the process (35 °C and 100 bar), the effect of the pVCL-ICA concentration was analyzed. Thus, 20, 25, and 30 mg/mL values were evaluated. Increasing the concentration could lead to (1) the supersaturation of the polymer, promoting NP nucleation and generating smaller-sized particles, or (2) higher condensation, producing larger NPs. The variations in the concentration of pVCL-ICA did not lead to significant differences between the NP sizes (Figure 3e,f). Despite this, the sample with the lowest concentration (20 mg/mL) exhibited the lowest size but also the highest encapsulation efficiency (see, again, Table 2). Based on the above results, this was selected as the optimum concentration.

Using the previously optimized T, P, and C parameters (35 °C, 100 bar, and 20 mg/mL), different ICA loads were evaluated. According to the results summarized in Table 2, the highest the ICA wt.%, the higher the encapsulation efficiency and particle size (see Figure 3g,h), with a spherical morphology in all cases (see, again, Table 2). Also, the DSC analysis indicated that at the highest ICA wt.%, a lower Tg value was observed, indicating the plasticizing effect of ICA on the Tg of pVCL. The three samples, NP5, NP10, and NP20, were used for the following studies.

### 3.3. Icariin In Vitro Release

**Figure 4:**
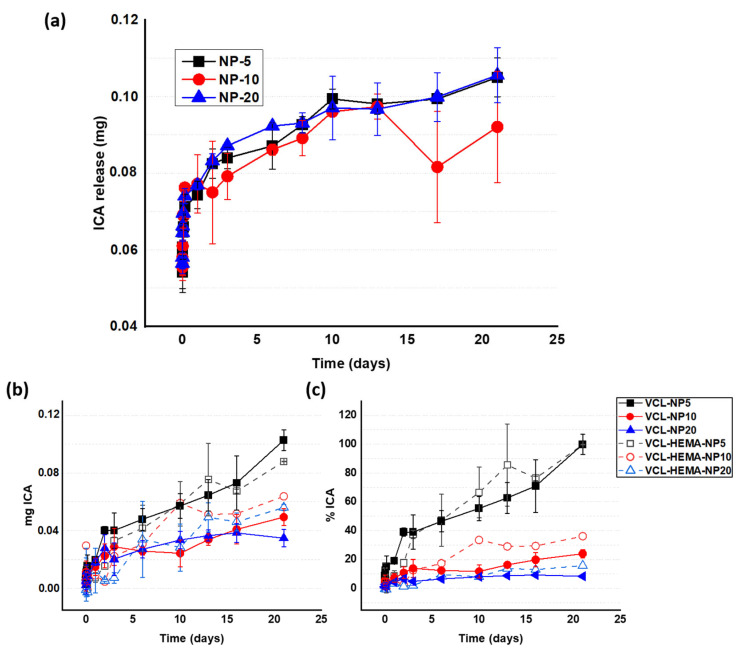
(**a**) ICA release profile (mg) from the nanoparticles (NP5, 10, and 20) at 37 °C. (**b**) ICA release from the hydrogels (mg) at 37 °C, using PBS as a medium. (**c**) Release percentage of ICA from the hydrogels, also at 37 °C.

The release from the NPs (NP5, NP10, and NP20) showed a burst effect at short times, followed by a slow and steady release (see Figure 4a). The three types of NPs show similar results, despite the differences in ICA content. This fact can be explained by the very low solubility of ICA (0.39 mg/mL in water) [37], which suggests that its release is self-regulated and is dependent on the medium turnover and not on the loading of the NPs. The low values of mass released may be due to the lower solubility of ICA in PBS compared to water, as seen in some cases when using PBS is mixtures with DMSO 10:1, showing a 0.1 mg/mL solubility [38].

The release from the hydrogels no longer shows this burst effect in Figure 4b; instead, a sustained release is observed, which is slightly dependent on both the ICA loading on the NP and the presence of HEMA. The sustained nature of the release must be associated with the double barrier that the drug has to overcome to be released into the medium; i.e., ICA is released from the NP and has to diffuse through the hydrogel network until it enters into the surrounding medium. The data for the different systems are close, so, in this case, self-regulation by the low solubility of ICA can also be suggested. The fact that NP5-loaded networks release more ICA than N10, and N10 more than N20 (loaded hydrogels), may be related to the differences in the NPs and their local behavior once they are loaded into the hydrogel. The sizes of the NPs increase in this order: NP5 < NP10 < NP20. In the context of low ICA solubility, these differences may explain why the ease of ICA release increases with the decreasing loading of ICA. In any case, Figure 4c, in which the data are plotted as a percentage, logically indicates that the higher the loading in the hydrogel with ICA, the longer the system can release ICA in a sustained fashion.

In Figure 4b,c, the presence of HEMA seems to have some influence on hydrogels loaded with NP10 and NP2O, for which the release with HEMA is higher than without HEMA. In the aforementioned low-solubility scenario, the local environment within the network (i.e., the presence of OHs) may be important. The polar interactions associated with the presence of OHs may locally increase the solubility of ICA, an aspect that has been reported when releasing hydrophobic drugs from hydrophilic polymer networks [39,40].

### 3.4. Osteoblastic Cell Sheet Transplants from pVCL Hydrogels Activated with ICA-NPs

Finally, as a proof of concept, the pVCL hydrogels activated with pVCL@ICA NPs (5, 10, and 20 wt.%), described in the previous section, were tested as an in vitro platform to produce differentiated osteoblastic cell sheets. As is well known, ECM plays a crucial role in tissue regeneration processes, especially in tissue injuries such as to bone or cartilage. The cytocompatibility of NPs was also preliminary evaluated, ensuring their safety as promoters of cellular differentiation into bone-type lineages (Supplementary Figure S7).

**Figure 5:**
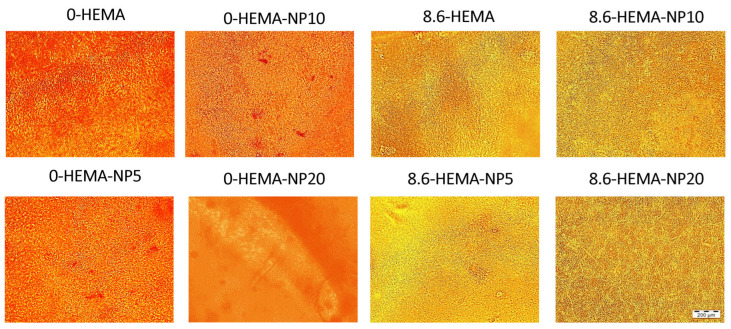
MC3T3−E1 osteoblastic cell cultures, proliferating over hydrogels at day 14 (scale bar: 200 µm).

In this approach, the MC3T3−E1 preosteoblastic model was seeded over NP-activated platforms and cultured for 14 days. Hydrogels without NPs (0−HEMA and 8−6-HEMA) were included in the study as controls. As can be appreciated from Figure 5, confluent cell monolayers were observed over all surfaces, with no differences in the proliferation states detected between samples.

Then, the hydrogels were placed into a new TCP well and the osteoblastic cell sheets were transplanted using a thermal stimulus, as previously described. The MC3T3−E1 osteoblastic transplants were cultured for an additional 8 days to attach and colonize the new surface. Finally, the cell cultures were fixed, and collagen type I production was evaluated through PicroSirius Red staining as a molecular marker for cell differentiation and organic ECM content analysis.

**Figure 6:**
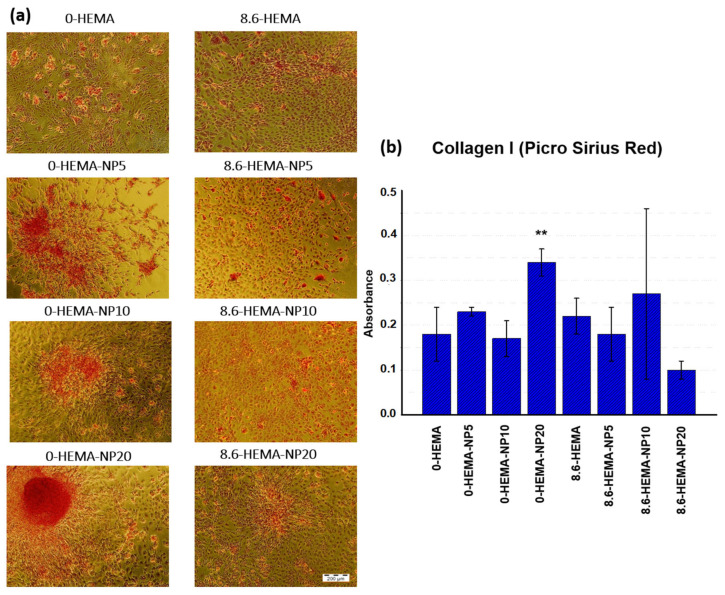
(**a**) PicroSirius Red staining (collagen I) from MC3T3−E1 osteoblastic transplants from hydrogels at day 8 after transplant; (scale bar: 200 µm). (**b**) PicroSirius Red quantification. Significant differences were indicated as follows: ** (*p* ≤ 0.01).

As can be observed in Figure 6, viable osteoblastic cell cultures were detected on all platforms. All transplanted samples showed positive staining for collagen type I, with a strongly focused signal in most confluent cell populations (0−HEMA−NP20). However, quantification analysis evidenced higher total collagen expression from the samples, including ICA NPs, such as in 0-HEMA−NP20 or 8.6−HEMA−NP10. In this scenario, the increased collagen content is related to osteoblastic cell proliferation and its differentiation state, both probably being promoted by NP-released ICA activity. It is noticeable that slow icariin release dynamics would favor cell differentiation, as the collagen I expression from 0−HEMA−NP20 and 8.6−HEMA−NP10 samples suggest, although no direct correlation is evidenced. Moreover, although a trend could also be identified for a specific upregulation from certain HEMA samples in comparison to their counterparts, no significant differences were observed. 

In this cell sheet engineering model, the transplant’s proliferative capacity should be conserved and combined with an optimal differentiation state for proper cell colonization and consolidation over the new surface. This tight equilibrium between cell proliferation and differentiation can explain some of the differences observed in this study, as collagen I content is as a result of both the total number of cells and their ECM secretion activity, related to the latter by the culture differentiation state. Probably, highly differentiated osteoblastic cultures can lose their ability to efficiently proliferate and colonize new surfaces. Conversely, 0−HEMA control platforms achieved osteoblastic transplantation and proliferation, but showed moderate collagen I expression due to their undifferentiated stage [41,42]. However, incorporating NPs containing ICA reverted this process, enhancing the differentiation and matrix deposition. As a result, the efficacy of 0−HEMA−NP20 hydrogels is remarkable in terms of conserving osteoblastic cultures in an optimal state for transplantation purposes.

## 4. Conclusions

In this work, p(VCL-co-HEMA) hydrogels with variable percentages in their composition were obtained and characterized using physicochemical analysis. Although no direct effect on the VPTT value was detected with the inclusion of HEMA, a higher transparency at the physiological temperature, as well as lower swelling at low temperature, was observed for those hydrogels containing higher concentrations. The cytocompatibility, transplant efficacy, and bone differentiation capacity of the p(VCL-co-HEMA) hydrogels were investigated using two different murine cell lines, namely, premyoblastic and preosteoblastic. All hydrogels supported these biological processes, with exceptional performance for 8.6% HEMA in terms of detachment ability. 

Subsequently, icariin-loaded pVCL nanoparticles were obtained using SAS-CO_2_ (5, 10, and 20 wt.% of ICA) and were included in hydrogels to assess their controlled release at the physiological temperature. After checking their efficacy as controlled icariin release platforms, a preosteoblastic model was cultured to test the influence of this biological factor on cell proliferation and differentiation. As a result, optimal formulations for hydrogels activated with icariin NPs were identified, both for 0 and 8.6% HEMA. Using this combined approach, viable osteoblastic sheets with high collagen type I content were transplanted and re-cultivated to evaluate their ability to colonize a new surface without losing an optimal ECM composition. 

Finally, these results open the possibility of a new clinical approach for treating critical bone and cartilage injuries, based on customized pVCL-based cell platforms with the possibility of encapsulating different drugs, a technique that can be used in combination with other implantation surgeries. In fact, the possibility of the encapsulation of different drugs or biological factors that not only refer to bone lineage by means of clean technology, added to the demonstrated capacity of their sustained release, represents an improvement over the intelligent platforms previously described by this research group, moving toward much more versatile and precise therapeutical systems. 

## Figures and Tables

**Table 1:** The VPTT and T_OT_ values, swelling data at 10, 37, and 55 °C, and transmittance at 25 and 37 °C of the X−HEMA hydrogels. The figures in the upper part show the swelling (S) variations with temperature seen in the prepared hydrogels (left) and the FTIR−ATR spectra of the representative samples (right).

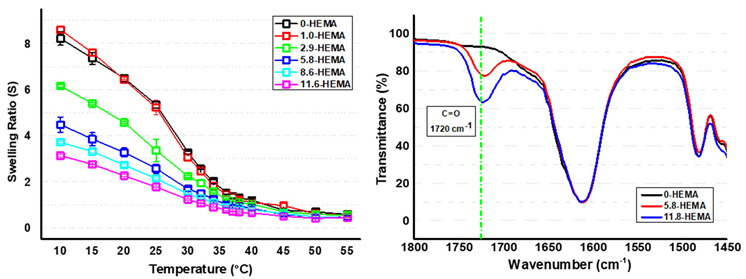								
Label	HEMA in Feed (Molar%)	VPTT (°C)	Swelling Ratio at (°C)			T_OT_ (°C)	Transmittance (%) at (°C)	
			10	37	55		25	37
0−HEMA	0-control	35	8.21 ± 0.30	1.35 ± 0.08	0.57 ± 0.05	33	68.7	15.9
1.0−HEMA	1.0	35	8.59 ± 0.42	1.32 ± 0.10	0.54 ± 0.09	34	79.3	45.2
2.9−HEMA	2.9	35	6.15 ± 0.07	1.14 ± 0.02	0.54 ± 0.05	34	84.3	56.4
5.8−HEMA	5.8	36	4.47 ± 0.33	0.98 ± 0.02	0.48 ± 0.06	34	93.3	78.7
8.6−HEMA	8.6	37	3.70 ± 0.05	0.88 ± 0.05	0.44 ± 0.04	35	93.3	92.1
11.6−HEMA	11.6	37	3.13 ± 0.06	0.69 ± 0.10	0.43 ± 0.06	37	96.7	96.6

**Table 2:** Proposed variables for the process for obtaining pVCL@ICA NPs: temperature, pressure, and solute–solvent ratio. The characteristics of the three systems prepared for the study under optimized conditions (NP5, NP10, and NP20) have been also included.

Parameter	Conditions	Comments and Labels	NPs Diameter (nm)	Tg (°C)	Encapsulation Efficiency (%)
Optimization					
Temperature, T (°C) ^a^	35	Selected T	540 ± 150	71	76
	40	-	No spherical morphology		
Pressure, P (bar)	100	Selected P	520 ± 130	71	76
	150	-	500 ± 230	62	72
	200	-	390 ± 120	63	84
pVCL-ICA/DMSO C (mg/mL) ^b^	20	Selected C	520 ± 130	71	91
	25	-	650 ± 210	57	89
	30	-	580 ± 180	58	85
pVCL@ICA-NPs prepared for the study					
ICA load (weight%)	5	NP5	450 ± 130	68	91
	10	NP10	520 ± 130	71	91
	20	NP20	590 ± 330	53	97

a: Pressure was 100 bars; b: 10 weight % of the ICA load.

## Data Availability

The data presented in this study are available in this article (and Supplementary Materials).

## Supplementary Materials

The following supporting information can be downloaded at: https://www.mdpi.com/article/10.3390/pharmaceutics16081063/s1, Figure S1. Turbidimetry (transmittance percentage vs. temperature) of the hydrogels. Both studies were carried out using a variable amount of HEMA (molar percentage) in PBS; Figure S2. Fluorescence images of the cell cultures on the hydrogels after 72 h of the seeding process (scale bar: 200 μm); Figure S3. Bright-field images of MC3T3−E1 premyoblastic cell cultures over hydrogels 30 days after the seeding stage (scale bar: 200 μm); Figure S4. (a) Bright-field images of PicroSirius Red staining (collagen fibers, PS-S) of MC3T3-E1 osteoblastic culture for 0, 2.9, 5.8, 8.6, and 11.6% HEMA hydrogels at 30 days (scale bar: 200 μm). (b) Collagen I quantification for cultures with the functionalized hydrogels. No significant differences (Student’s *t-*test) were detected; Figure S5. (a) dsDNA quantification of MC3T3−E1 cultures proliferating on HEMA hydrogels at 48 and 120 hours after cell seeding. (b) Bright field images of MC3T3−E1 cultures 72 h after transplantation stage and quantification of metabolic activity by AlamarBlue; (scale bar: 200 µm); Figure S6. ^1^H−NMR spectra of the synthesized pVCL; Figure S7 (a) Bright-field images of MC3T3-E1 cell culture proliferation at 96 and 168 h. (b) Metabolic activity (Alamar Blue) of MC3T3-E1 proliferation at 96 and 168 h. (c) Bright-field images of PicroSirius Red staining (collagen fibers, PS-S) of MC3T3−E1 osteoblastic culture for pVCL@ICA NPs at 26 days (scale bar: 200 μm).
