# Supplementary material for: Osteoblastic Cell Sheet Engineering Using P(VCL-HEMA)-Based Thermosensitive Hydrogels Doped with pVCL@Icariin Nanoparticles Obtained with Supercritical CO2-SAS
Source: Pharmaceutics. 2024 Aug 13;16(8):1063. doi: 10.3390/pharmaceutics16081063 (PMC11359487; doi:10.3390/pharmaceutics16081063)
Supplement: Supplementary file 1 [file pharmaceutics-16-01063-s001.zip › pharmaceutics-3063177-supplementary.pdf]

Supplementary Material

Osteoblastic Cell Sheet Engineering Using P(VCL-HEMA)-Based Thermosensitive Hydrogels Doped with pVCL@Icariin Nanoparticles Obtained with Supercritical CO_2-SAS

Rubén García-Sobrino [1,2,3], Isabel Casado-Losada [1,2], Carmen Caltagirone [1,2], Ana García-Crespo [1,2], Carolina García [1], Juan Rodríguez-Hernández [1], Helmut Reinecke [1], Alberto Gallardo [1], Carlos Elvira [1,*] and Enrique Martínez-Campos [1,2,*]

1 Polymer Functionalization Group, Departamento de Química Macromolecular Aplicada, Instituto de Ciencia y Tecnología de Polímeros-Consejo Superior de Investigaciones Científicas (ICTP-CSIC) Calle Juan de la Cierva, n◦3, 28006 Madrid, Spain; ruben.sobrino@urjc.es (R.G.-S.); carolina@ictp.csic.es (C.G.); hreinecke@ictp.csic.es (H.R.)

2 Group of Organic Synthesis and Bioevaluation, Instituto Pluridisciplinar, Universidad Complutense de Madrid (UCM), Associated Unit to the ICTP-IQM-CSIC, Paseo Juan XXIII, n◦1, 28040 Madrid, Spain

3 Department of Applied Mathematics, Materials Science and Engineering and Electronic Technology, Universidad Rey Juan Carlos, Calle Tulipán s/n, 28933 Móstoles, Spain

* Correspondence: celvira@ictp.csic.es (C.E.); e.martinez.campos@csic.es (E.M.-C.); Tel.: +34-91-562-29-00 (C.E. & E.M.-C.)

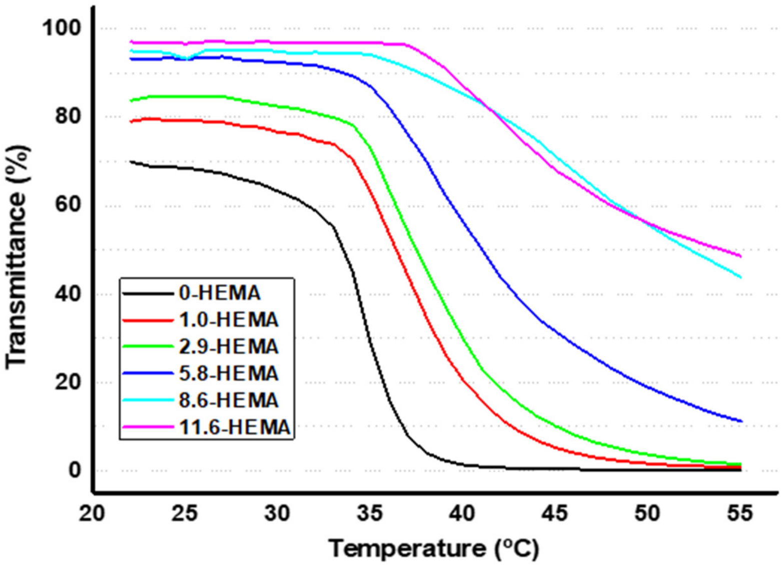

Figure S1. Turbidimetry (transmittance percentage vs. temperature) of the hydrogels. Both studies were carried out using a variable amount of HEMA (molar percentage) in PBS.

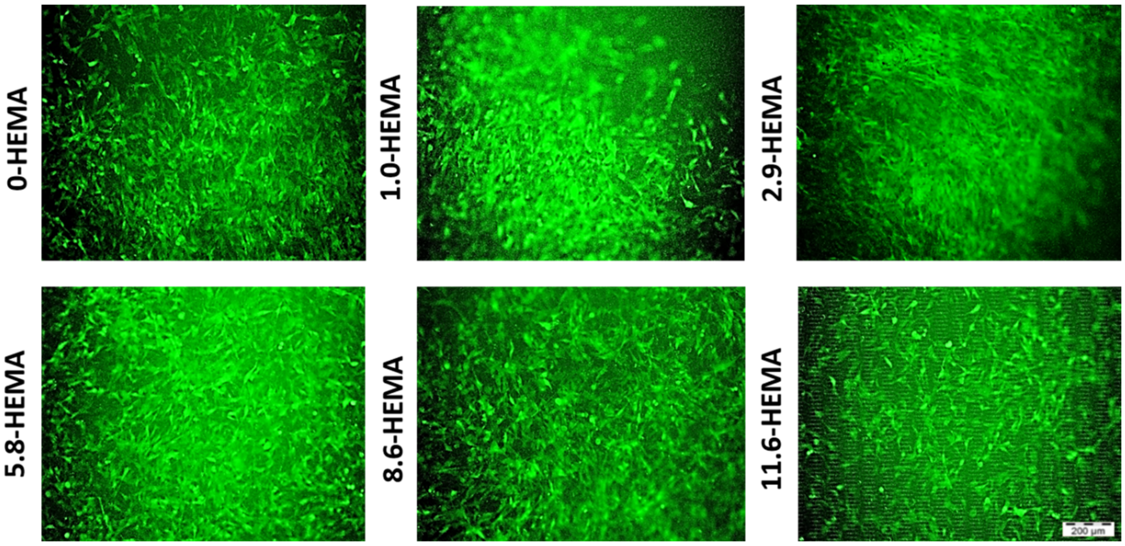

Figure S2. Fluorescence images of the cell cultures on the hydrogels after 72 h of the seeding process; (scale bar: 200 μm).

Osteoblastic cell differentiation on pVCL-co-HEMA hydrogels

To evaluate the ability of HEMA groups as a positive stimulant for bone tissue, preosteoblastic cell line MC3T3-E1 was selected. This cell line conserves proliferating capacity in the absence of bone differentiation stimuli. On the other hand, in the presence of differentiation agents (such as defined media or other active biological factors), osteoblasts upregulate bone extracellular matrix secretion, containing mainly collagen type I amongst other components. For this study, it is necessary to highlight that the molar percentage of 1% HEMA has not been included due to the previous results. Both in the physicochemical tests and the cell proliferation and detachment analyses, this percentage of HEMA hardly showed any differences respect to the control hydrogel.

As described in **section 2.5.5** of the manuscript, osteoblastic MC3T3-E1 culture was first cultured in proliferation medium on the hydrogels to promote cell growth and afterwards, changed to differentiation medium. **Figure S-3** shows that all the proposed hydrogels exhibited proliferative capacity, and therefore, cytocompatibility with this cell line.

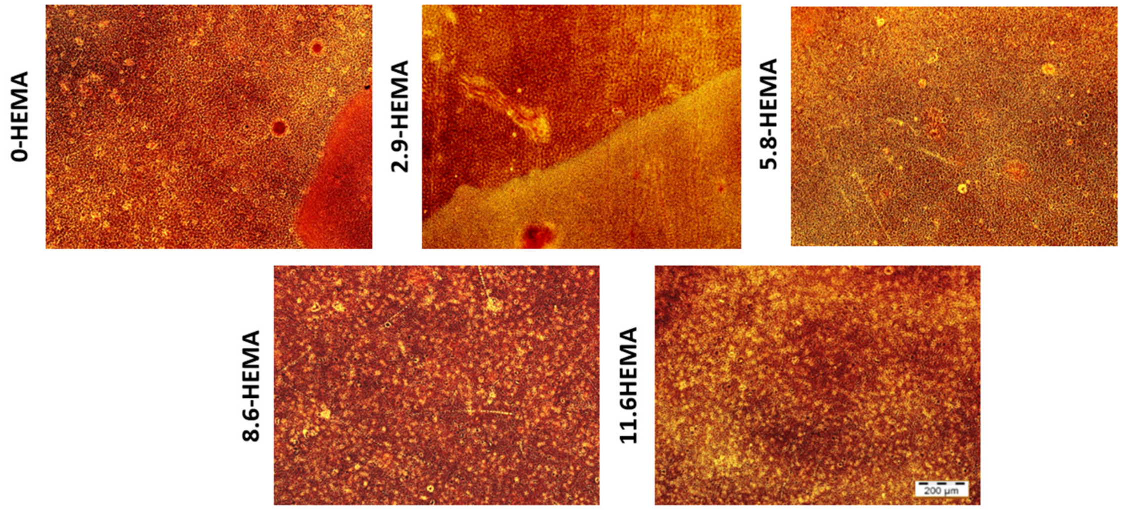

Figure S3. Bright-field images of MC3T3-E1 premyoblastic cell cultures over hydrogels 30 days after the seeding stage; (scale bar: 200 µm).

Then, collagen type I was selected to study organic bone matrix secretion from MC3T3-E1 cultured onto the hydrogels. Collagen fibers after Picro Sirius staining were evidenced, demonstrating that samples allowed osteoblastic differentiation and subsequent bone matrix formation (**Figure S4 (a)**). In addition, collagen staining was quantified according to the HEMA molar percentage (**Figure S4 (b)**). In this study the presence of HEMA did not show any significant differences in terms of collagen secretion with respect to the control hydrogel.

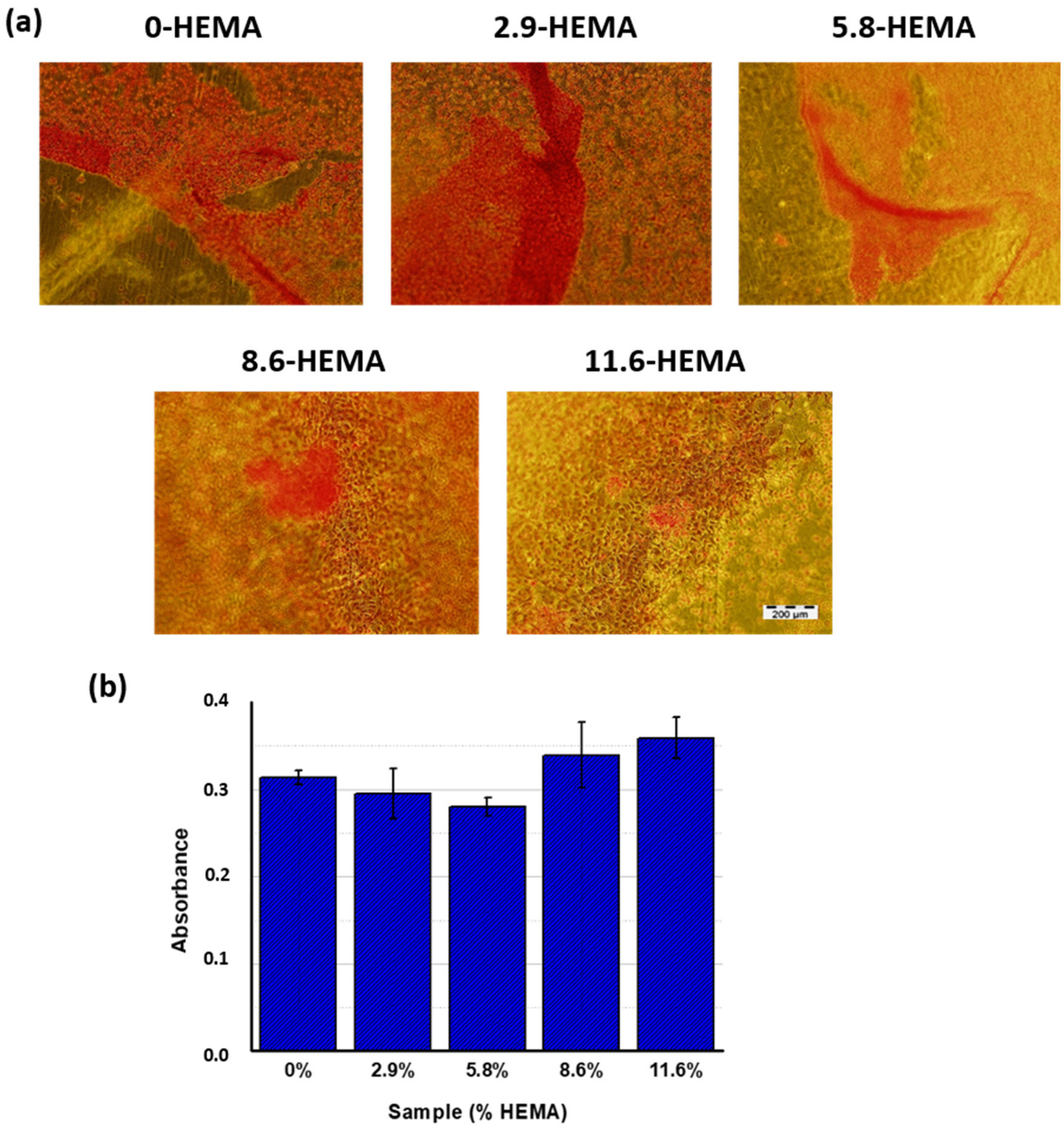

Figure S4. (a) Bright-field images of Picro Sirius Red staining (collagen fibers, PS-S) of MC3T3-E1 osteoblastic culture for 0, 2.9, 5.8, 8.6 and 11.6 % HEMA hydrogels at 30 days; (scale bar: 200 µm). (b) Collagen I quantification for the cultures with the functionalized hydrogels. No significative differences (t-student) were detected.

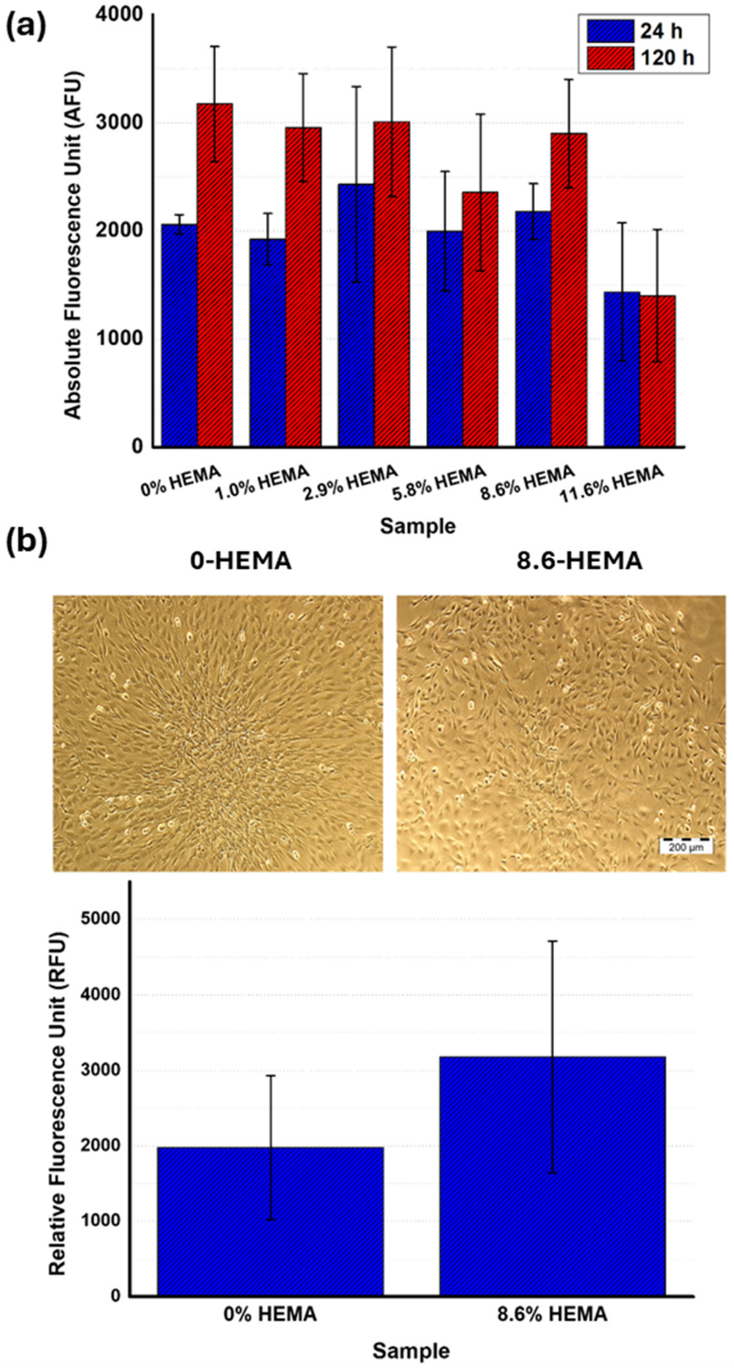

Figure S5. (a) dsDNA quantification of MC3T3-E1 cultures proliferating on HEMA hydrogels at 48 and 120 hours after cell seeding. (b) Bright field images of MC3T3-E1 cultures 72 h after transplantation stage and quantification of metabolic activity by AlamarBlue; (scale bar: 200 µm).

In **Figure S5 (a)**, it can be appreciated that a slight upregulation in cell proliferation with intermediate HEMA percentages is observed at 48 hours, especially for 2.9-HEMA. However, these differences are attenuated at 120 hours, where almost all samples showed similar levels of

DNA content. Surprisingly, 11.6-HEMA hydrogels showed a reduced level of osteoblastic cell proliferation both at 48 and 120 hours.

Additionally, a short-term transplant of MC3T3-E1 cell cultures using 0-HEMA and 8.6-HEMA was done after 120 h of proliferation (37 ºC and 5% CO_2). **Figure S-5 (b)** shows a modest improvement of transplant efficacy with 8-6-HEMA 72 hours after transplantation, confirming our previous observations.

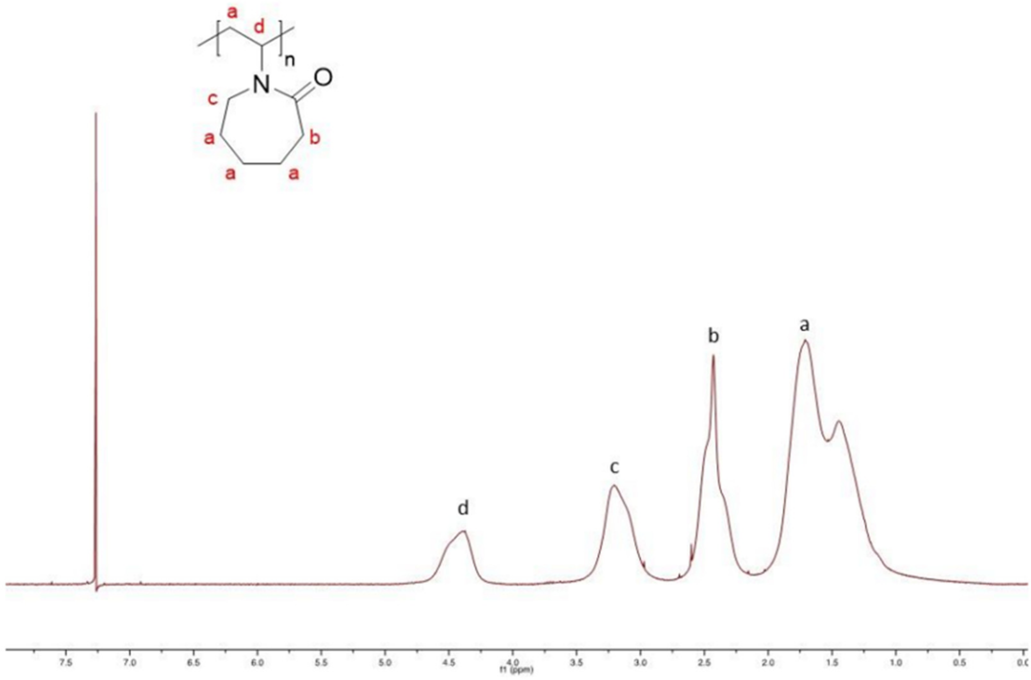

Figure S6. ^{1}H-NMR spectra of the synthesized pVCL.

Preliminary biological evaluation of pVCL@ICA NPs

Cytocompatibility and osteogenic properties of the pVCL@ICA nanoparticles were preliminary evaluated. First, MC3T3-E1 were seeded at a density of 1×10^4 cells/cm^2 on treated TCP well plate. Subsequently, and after NPs sterilization, they were dissolved first in PBS at a concentration of 10^{-3} mol/L, and later on incorporated to α-MEM proliferation medium, at a final concentration of 10^{-5} mol/L. Cells were cultured under proliferation conditions for 5 days, and switched to α-MEM medium differentiation for additional 21 days, with media changes every 72 h. Micrographs and metabolic activity quantification with Alamar Blue kit´s analysis were performed to confirm cell viability of pVCL@ICA (**Figure S-7 (a)** and **(b**) at 96 and 168 h. Based on the mentioned, no signs of cytotoxicity were detected in osteoblastic cell sheets in contact with NPs. Similarly, the osteoinductive capacity of the prepared NPs was preliminarily analysed. In this sense, the evaluation developed with Picro Sirius Staining showed qualitatively from images the formation of bone tissue in the presence of the prepared drug (see **Figure S-7 (b)**).

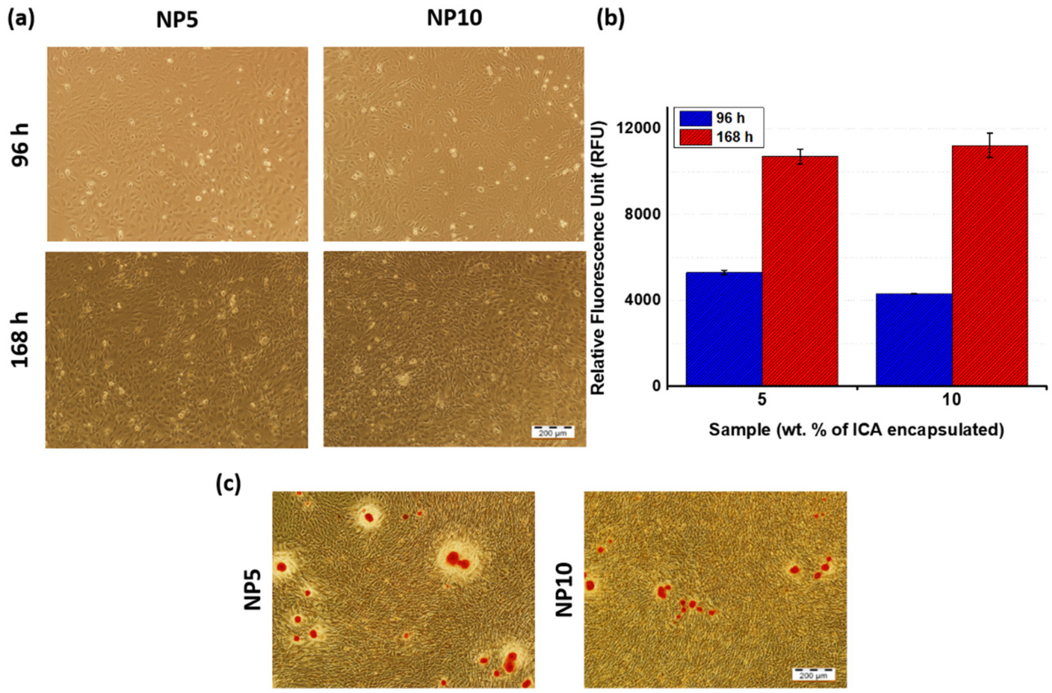

Figure S7. (a) Bright field images of MC3T3-E1 cell culture proliferation at 96 and 168 h. (b) Metabolic activity (Alamar Blue) of MC3T3-E1 proliferation at 96 and 168 h. (c) Bright-field images of Picro Sirius Red staining (collagen fibers, PS-S) of MC3T3-E1 osteoblastic culture for pVCL@ICA NPs at 26 days; (scale bar: 200 µm).
